# Regional accents modulate perspective in geographical space

**DOI:** 10.1007/s00426-021-01607-1

**Published:** 2021-10-18

**Authors:** Enrique García-Marco, Itatí Branca, Dolores Castillo, Inmaculada León, David Beltrán, Manuel de Vega

**Affiliations:** 1 Edificio NEUROCOG, Universidad de La Laguna, Campus de Guajara, 38200 La Laguna, Canary Islands Spain; 2 Instituto Universitario de Neurociencia (IUNE), La Laguna, Spain; 3 Universidad Europea de Canarias (UEC), La Orotava, Spain; 4 Universidad Nacional de Educación a Distancia (UNED), Madrid, Spain; 5 Universidad Nacional de Córdoba, Córdoba, Argentina; 6 Centro de Estudios Multi-disciplinarios en Sistemas Complejos y Ciencias del Cerebro (CEMSC3), Universidad Nacional de San Martín (UNSAM), Buenos Aires, Argentina

## Abstract

In this study, participants listened to first-person statements that mentioned a character who was approaching a geographical location close to (Tenerife, Canary Islands) or distant from the participant (Madrid, Spanish peninsula), pronounced with either the participants' local or a distal regional accent. Participants more often judged approaching statements as coherent when they refer to a close place pronounced with local accent or refer to a distant place with distal accent, rather than when they refer to a close place with distal accent or to a distant place with local accent. These results strongly suggest that the local accent induces listeners to keep their own geographical perspective, whereas the distal accent determines shifting to another’s perspective. In sum, a subtle paralinguistic cue, the speaker’s regional accent, modulates the participants’ geographic perspective when they listen to identical first-person sentences with approaching deictic verbs.

## Introduction

Humans can communicate effectively about objects' spatial location in a variety of situations. When processing narratives, we make inferences that allow us to know under which conditions speakers refer to their own point of view (self-perspective) or intend a shift to another person's point of view (other-person perspective) when understanding these sentences. These perspective effects are modulated by various factors, including individual differences (Hartung et al., 2017). The present study aimed to extend prior research on narrative perspective by investigating how a subtle paralinguistic cue, namely the regional accent, during narrative comprehension, influences the interpretation of an utterance.

In face-to-face conversations, speakers frequently use their current spatial and temporal location as a 'deictic center', namely, their' here—now—I' that serves as a framework for communication about the current situation (Zubin, & Hewitt, 1995; Duchan et al., 1995; Bühler, 1982). In this regard, most languages have a repertoire of deictic words, such as the pronouns 'I', 'you', and 'she/he' referring to the speaker, the addressee, or another person, respectively. Also, verbs such as 'come' or 'bring' denote motion towards the deictic center (or speaker's location), and verbs such as 'go' or 'send' mark motion away from it. For instance, if someone tells us 'I am going to bring the book tomorrow', we assume that 'I' refers to the current speaker, 'bring' designates transporting the book to the place where we are having the conversation, and 'tomorrow' refers to the day after the speaking time. In conversational settings, the effective understanding of this kind of utterance relies on the current situation that the speaker and the addressee share. By contrast, when we are reading narratives, the deictic center is neither the author's nor the reader's spatio-temporal framework. Instead, it is a virtual deictic center in the narrative world (Black et al., 1979; Rapaport et al., 1994).

The role of deictic pronouns on perspective-taking has been analyzed in several studies. In a single-pulse transcranial magnetic stimulation (TMS) experiment, Papeo et al. (2011) found that subjects increased motor evoked potentials (MEPs) when they read first-person action verbs compared to non-action verbs. However, when third-person action and non-action verbs were presented, there was no significant differential modulation of MEPs. Papeo et al. (2011) interpreted these results as proof that the grammatical first-person induce self-perspective, modulating motor simulations in action verbs. In another study, Brunyé et al. (2009) explored the role of pronouns in modulating the reader's adopted perspective. The task consisted of reading sentences including a first-, second-, or a third-person pronoun (I, you, or he) and verifying whether a picture matched or mismatched the action in the sentence, regardless of the picture perspective (images captured either an actor's or an observer's perspective). The pronoun 'you' clearly induced a self-perspective, as verification was faster when the picture depicted an action matching the reader's point of view rather than an external one. The verification times for 'I' sentences, however, showed more complex patterns. In natural contexts, when we listen to first-person utterances, they usually come from an interacting partner talking about her/himself ('I think that…'). In contrast, when we read first-person narratives, we understand them as referring to the story protagonist rather than the self. In experimental settings, isolated first-person pronouns ('I', 'me', 'mine') have frequently been used as a tool for studying self-perspective and self-awareness in the language (Shi et al., 2011; Zhou et al., 2010), or in very short sentences strictly related to personal states or traits (e.g., 'I am happy') (Esslen et al., 2008; Johnson et al., 2002). However, the presence of a brief narrative describing a protagonist who uses the pronoun 'I' induces participants to shift to the other-person perspective (Brunyé et al., 2009). In sum, the pronoun 'I' elicits self- or other-perspective depending on contextual factors.

Unlike in face-to-face communication, in narratives readers usually make a deictic shift, disregarding their own deictic center to assume the character's perspective in the narrative world. Therefore, deictic words like 'I', 'come', or 'tomorrow' would refer respectively to the fictitious speaker, a motion toward her, and the day after the narrative present. The cognitive processes of a deictic shift are complex; they have been theoretically discussed (Galbraith, 1995; Segal, 1995) and empirically explored in a few studies (Black et al., 1979; García-Marco et al., 2016; Zwaan, 2004). However, an ERP study performed in our laboratory demonstrated that in some circumstances, readers could use their own geographical deictic center to understand deictic expressions (de Vega et al., 2015). Participants were asked to read short paragraphs with deictic verbs describing motions toward or away from the deictic center. The paragraphs were written in the second person, describing a situation in which 'you' met another character who tells you she/he is 'coming to', 'going to', or 'being in' either the participant's own geographical location or a distant place. For example, "A few days ago, you met a young architect. He told you that he had come to [local place] [distant place]…" The ERPs were sensitive to the congruence of motion sentences with the readers' geographical place. For instance, participants living in Tenerife showed a larger N400, an electrophysiological marker of semantic coherence (Kutas & Hillyard, 1980, 1984), when they read 'She came to Barcelona' (distant place) than when they read 'She came to Tenerife' (local place). Also, deictic sentences referring to the local place elicited larger N1, P2, and P3 components, frequently associated with self-relevance (Shi et al., 2011; Zhou et al., 2010), than those referring to distant places. Interestingly, these effects occurred in sentences with deictic verbs ('coming' and 'going') but not with stative verbs ('being'). The authors concluded that, in the absence of an explicit narrative deictic center, subjects used their own deictic center by default (de Vega et al., 2015).

According to previous literature, 7-year-old British children are able to categorize unfamiliar regional accents as differing from their native accents (Floccia et al., 2009), and 5-year-old Spanish-speaking children discriminate among ten different Spanish regional accents (Arango, 2016). In addition, listeners are able to extract from features of the speaker’s voice, implicit contextual knowledge (Lattner & Friederici, 2003). These empirical facts are relevant for the present study, because we wanted to induce perspective effects based on paralinguistic features of the speaker’s voice that is the regional accent. The present study manipulates linguistic materials to induce geographical-perspective effects, like in de Vega et al.'s (2015) study. Specifically, this study aims to explore whether the speaker’s regional accent could induce that the listener takes the self- or the other-deictic perspective. The participants listened to first-person contexts, in which a critical sentence described a character's motion toward their own geographical place (Tenerife, Canary Islands) or toward a distant geographical place (Madrid, Spanish mainland). Notice that, unlike in de Vega et al.’s study, our experimental contexts described character's motions using only proximal (e.g., ‘come’) but not distal deictic verbs (e.g., ‘go’), and thus the intended perspective effects cannot rely on the directionality of the verb but only on the paralinguistic cue of accent. This could avoid a potential problem in de Vega et al.’s, in which geographical perspective effects could be explained to some extent in terms of word co-occurrence. Thus, people who live in Tenerife more frequently listen to 'coming to Tenerife' than ‘going to Tenerife’ phrases, and consequently, they would need less effort to understand the former than the latter (see de Vega et al., 2015 for a discussion of this issue). In this study, we could rule out this explanation, since geographical perspective effects only rely on the speaker’s accent and cannot be confused with the "statistical" properties of the linguistic materials.

The sentences were pronounced by speakers either with the local accent or with a distinctive accent from the distant region. Given that Tenerife's local accent and Madrid's distant regional accent (Castilian) differ substantially, we verified that Spanish adults identify them easily. The hypothesis was that the local accent would induce the default geographical self-perspective, whereas the distant-region accent would induce a deictic shift to the speaker's geographical perspective. The critical sentences described the character's motion using two Spanish proximal deictic verbs: 'venir' (herein 'come') already explored in previous studies (de Vega et al., 2015; García-Marco et al., 2016), and included for the first time 'traer' ('bring'). The verb 'come' is intransitive, referring just to a person's body displacement; by contrast, 'bring' is a transitive verb that implies an object's displacement generally associated with a person's motion. The transitive verbs are semantically more complex than the intransitive verbs, involving implicit causality in the deictic motion. Thus, 'bring' can be paraphrased as "cause to come" (Fillmore, 1975; Levinson, 1996; Miller & Johnson-Laird, 1976). In spite of that, we expect that both the transitive and the intransitive verb will be equally sensitive to the regional accent to establish a geographical perspective.

Concerning the role of the grammatical person in perspective-taking, we have seen above that second-person sentences induce the reader or listener to use self-perspective, and third-person narratives guide readers to take the other-person perspective (de Vega et al., 2015; García-Marco et al., 2016). By contrast, first-person sentences are more ambiguous, as they could be interpreted as referring to the participant's self or to an external narrator's self, depending on specific contextual cues. For this reason, we choose first-person sentences, which presumably will be very sensitive to the regional accent to induce either the perspective of the self or another person's perspective. The participants judged whether the statements were coherent, and their responses were collected to infer their perspective choice. That is, we predict that “movement to close place with local accent”, and “movement to distant place with distant accent” will be judged more often as coherent than “movement to close place with distant accent” and “movement to distant place with local accent”.

## Methods

### Participants

Forty Spanish-speaking undergraduates (31 females, all between 18 and 27 years of age) gave informed consent and received course credit for their participation. All reported normal hearing and no neurological or neuropsychological disorder. An important inclusion criterion for the study was that all the participants have been living on the island of Tenerife (local place) for at least the last 2 years, and none had lived in Madrid (distant place) for more than 2 months. The experiment was performed in the town of La Laguna in Tenerife.

### Materials and design

A total of 40 experimental statements were presented aurally in Spanish, in a 2 Accent (Local/Distal) × 2 Geographical place (Close/Distant) repeated measure factorial design. The regional accents were from Canary Islands (local) and Castilian from Madrid (distal), which are easily distinguished by the Spaniards. The Close geographical places were the island (Tenerife), and the region (Canary Islands) where the participants were performing the experiment task, and the Distant geographical places were a city on the mainland (Madrid) and the mainland itself (Peninsula). The geographical distance between Tenerife and Madrid is about 1092 miles in a straight line, and the closest point between Tenerife and the Peninsula is approximately 800 miles. Each context included a first-person introductory sentence describing an encounter with another character, followed by a sentence referring to the character approaching a geographical location, and the object or goal for this motion. Two proximal deictic verbs were used to describe the character’s approaching motion. Specifically, half of the items in each condition included the transitive verb ‘traer’ (bring), and the remaining the intransitive verb ‘venir’ (come). Remarkably, the contexts did not provide any explicit information on the place where the participant was; the perspective-ambiguous pronoun “I” referred to the implicit narrator, and only the secondary character's motion towards a geographical goal was mentioned. In addition, 70 filler paragraphs with no geographical displacement were included, 15 of them semantically incoherent. Examples of the materials are presented in Table 1.

**Table 1:** Materials and fillers

Example with verb ‘TO BRING’	
Introduction	On Sunday I met a friend who hates traveling with lots of luggage (El domingo me encontré con una amiga que odia viajar con mucho equipaje)
Bring to close place	and because of that she had brought only a briefcase to the Canary Islands (y por eso había traído sólo un maletín a Canarias)
Bring to distant place	and because of that she had brought only a briefcase to the Peninsula (y por eso había traído sólo un maletín a la Península)
Example with verb ‘TO COME’	
Introduction	Yesterday I met a painter who specializes in landscape painting (Ayer conocí a un pintor especializado en pintura paisajística)
Come to close place	and he told me that he had come to make an exhibition in La Laguna (y me dijo que había venido a hacer una exhibición en La Laguna)
Come to distant place	and he told me that he had come to make an exhibition in Madrid (y me dijo que había venido a hacer una exhibición en Madrid)
Example of semantically coherent fillers	
Introduction	Ana was going to participate in the amateur singers contest, but just the day before she got hoarse (Ana iba a participar en el concurso de cantantes aficionados, pero justo el día anterior se quedó totalmente afónica)
Example of semantically incoherent fillers	
Introduction	I like vegetarian food, so I went to the greengrocer. Once there, I bought a kilo of oranges and two free-range chickens (Me encanta la comida vegetariana, de modo que fui a la verdulería. Una vez allí, compré un kilo de naranjas y dos pollos camperos)

Examples of the four versions of the materials combining the two deictic verbs with local and distant places, and a semantically coherent and incoherent filler in a literal English translation and in their original Spanish

### Procedure

Participants were randomly assigned to one of the four lists. The presentation of the stimuli and the recording of the participant's responses and response times were controlled by E-Prime software (version 2.0.10.242, Psychology Software Tools; Schneider et al., 2002). The paragraphs of the list were presented in random order, counterbalanced across subjects.

Participants were sat in front of a computer screen in a quiet room and received written instructions to listen carefully to phrases presented, and to indicate at their own discretion whether the phrase heard is coherent or not when the screen turns green. Response speed and accuracy were both recommended. Participants judged the passage coherence by pressing the "yes" or "no" assigned button, and the responses were collected for further analysis. The instructions for making the coherence judgments were intentionally generic because we wanted to test participants' intuitive and spontaneous responses rather than guide them. Moreover, the filler paragraphs aimed to prevent participants from focusing exclusively on geographic information, reinforcing the generic and open character of the coherence judgment (see Table 1). A training phase of a block of five phrases preceded the test phase.

All analyses were conducted using R software (R Core Team, 2019) through ULLRToolbox (Hernández-Cabrera & Betancort, 2019).

## Results

The probability of “yes” coherence judgments and judgment times per condition were submitted to Accent (Local vs. Distal) × Geographical place (Close vs. Distant) repeated measures ANOVA. Even though the data does not match normality criteria (Kolmogorov–Smirnov test showed *p* values below < 0.003), a parametric analysis was selected since RM ANOVA allows reporting the 2 × 2 interaction and the power of the effects. In addition, the sample was large enough as there were up to 40 observations per group and no extreme outliers. Moreover, the t-test can support moderately skewed distributions (Le Cessie et al., 2020) and even largely skewed distributions (Fagerland, 2012), although it was not the case with our data.

### Coherence judgments

**Fig. 1:**
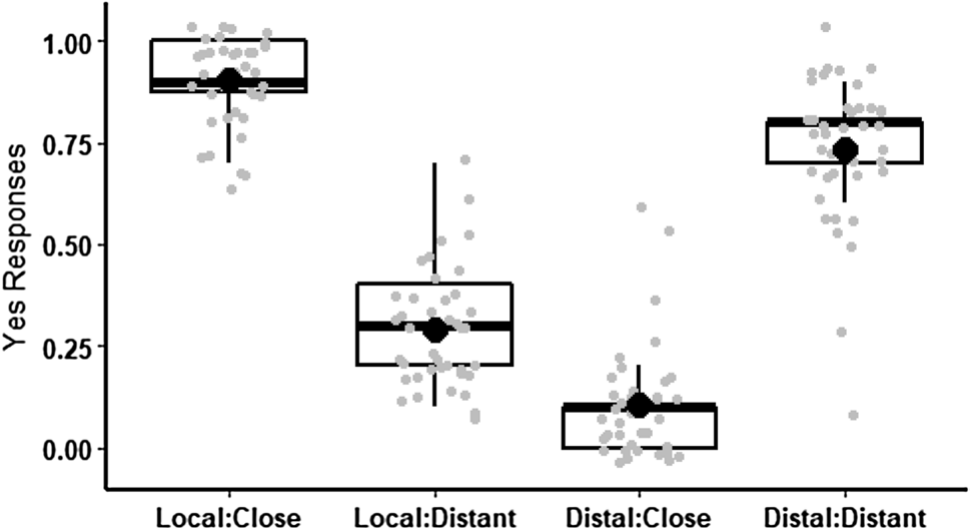
*F*^1^ ANOVA for probability of “Yes” responses. Probability of “yes” responses in the coherence judgment task for each experimental condition, combining Accent (Local vs. Distal) and place name (Close vs. Distant). The dots represent mean per condition and subject, the big dots represent mean per condition

A main effect of Accent was also found (*F*^1^ (1, 39) = 24.8, *p* < 0.001, $\eta_{p}^{2}$ = 0.39, *F*^2^ (1, 39) = 53.6, *p* < 0.001, $\eta_{p}^{2}$ = 0.58. The local Canary Island accent was more likely to be judged as coherent (*M* = 0.60, SD = 0.13) than the Madrid accent (*M* = 0.42, SD = 0.14). However, this effect is modulated by the important and robust Accent × Geographical place interaction (*F*^1^ (1, 39) = 174.55, *p* < 0.001, $\eta_{p}^{2}$ = 0.82, *F*^2^ (1, 39) = 621.07, *p* < 0.001, $\eta_{p}^{2}$ = 0.94) (see Fig. 1 for *F*^1^ ANOVA). The pairwise comparisons with Kenward–Roger´s degrees-of-freedom considering the two factors (Accents and Geographical Place), method and Hochberg correction for multiple comparisons showed that “Local accent and Close place” (*M* = 0.91, SD = 0.16) was judged as more coherent than “Distal accent and Close place” (*M* = 0.11, SD = 0.18), (*t* (117) = 16.72, *p* < 0.001) and “Local accent and Distant place” (*M* = 0.29, SD = 0.31), (*t* (117) = 12.85, *p* < 0.001). Finally, “Distal accent and Distant place” was judged as more coherent than “Local accent and Distant place” (*t* (117) = 9.25, *p* < 0.001), and “Distal accent and Close place” (*t* (117) = 13.12, *p* < 0.001). In other words, the consistency of accent and place (local-close and distal-distant) induced significantly more ‘coherent’ responses.

Concerning the coherence judgment times, the critical Accent x Geographical place interaction was not significant (*F*^1^ (1, 39) = 1.73, *p* = 0.19, $\eta_{p}^{2}$ = 0.042, *F*^2^ (1, 39) = 1.93, *p* = 0.17, $\eta_{p}^{2}$ = 0.047), although we found a Main effect of Geographical Place (*F*^1^ (1, 39) = 16.85, *p* < 0.001, $\eta_{p}^{2}$ = 0.30, *F*^2^ (1, 39) = 18.90, *p* < 0.001, $\eta_{p}^{2}$ = 0.33), being faster the judgments for close than distant places.

The main hypotheses tested in the study concern the basic design Accent x Geographical place, with the Type of Verb collapsed. Nonetheless, a complementary analysis was performed to check whether the control variable Type of Verb (Transitive vs. Intransitive) also modulated performance. To this aim an ANOVA Type of verb × Accent × Geographical place was performed. The results did not show significant main effect of Type of Verb (*F*^1^ (1, 39) = 0.06, p = 0.81, $\eta_{p}^{2}$ = 0.001, *F*^2^ (1,38) = 0.04, *p* = 0.84, $\eta_{p}^{2}$ = 0.001, nor its interaction with Accent (*F*^1^ (1, 39) = 0.06, *p* = 0.07, $\eta_{p}^{2}$ = 0.08, *F*^2^ (1,38) = 2.28, *p* = 0.14, $\eta_{p}^{2}$ = 0.06), Geographical Place (*F*^1^ (1, 39) = 0.005, *p* = 0.94, $\eta_{p}^{2}$ = 0.0.0001, *F*^2^ (1, 38) = 0.003, *p* = 0.95, $\eta_{p}^{2}$ = 0.001) or the three-way interaction (*F*^1^ (1, 39) = 0.18, *p* = 0.67, $\eta_{p}^{2}$ = 0.005, *F*^2^ (1,38) = 0.12, *p* = 0.73, $\eta_{p}^{2}$ = 0.003).

We conducted the post-hoc power analysis via G*Power (version 3.1.9.2) (Erdfelder et al., 1996) to define our Power by using the existing effect size of interaction. We found the interaction existing effect gives 100% power for Yes responses with our sample of 40 participants and therefore, excludes the possibility that the experiment is underpowered.

These results showed that the participants listening to first-person deictic paragraphs rely on the regional accent as a cue to establish the deictic center, indicating that the participant's regional accent has a privileged status as a cue for geographical perspective taking.

## Discussion

The present study provides insights into how listeners build spatial representations from minimal narratives. The narratives used here had no explicit information on the narrator's surroundings but did provide deictic cues that could prompt participants to take a geographical perspective. First, they used the first grammatical person which, depending on the context, could induce participants to use self or others perspective (e.g., Brunyé et al., 2009). Second, the narratives described motions to geographical places using proximal deictic verbs (‘come’ and ‘bring’), which are deictically marked and could also induce perspective-taking. We hypothesized that, in the absence of any explicit deictic center, participants would rely on, or be aligned with, their own geographical here-and-now to understand the deictic paragraphs. Moreover, we explored the new hypothesis that listeners could use the speaker's regional accent as a cue either to keep their own perspective or to shift to the other person's perspective. Finally, as a secondary goal, we also tested whether the perspective effects occur with transitive ('bring') and intransitive deictic verbs ('come').

In a nutshell, we obtained several remarkable results. Participants listening to first-person deictic paragraphs with proximal deictic verbs rely on the regional accent as a powerful cue to establish the deictic center. That is, statements that describe an approach to participants’ own geographical location were more often judged to be coherent when spoken with the local rather than the distal accent, suggesting self-perspective. In contrast, statements that mention an approach to a distant location were more often judged to be coherent when spoken with the distal rather than the local regional accent, suggesting a shift to another-person’s perspective. The results were similar for both transitive and intransitive deictic verbs, indicating that the modulation of perspective induced by regional accent was equally robust despite the fact that these verbs differ in semantic complexity. One relevant point to consider is that we found significant interactions for coherence judgments and not for Reaction Times, which could somehow limit the interpretation of the robustness of the effect. However, the null effects on RT might be justified because participants made coherence evaluation under the same repetitive sequence, and they could have mentally decided while listening to the item before having the opportunity to respond prompted by the green screen cue. This fact would favor quick answers and delude the effects on RT.

A novelty in this experiment is that the perspective effects occur here with large-scale spatial information, whereas most studies on language and space are based on small-scale layouts such as objects placed on a table or in a room (Avraamides & Carlson, 2003; Bower & Morrow, 1990; de Vega & Rodrigo, 2001; Franklin & Tversky, 1990; Hatzipanayioti et al., 2016). Notice, however, that the space around us could be organized into hierarchical categories such as this table, this room, this building, this town, this region, or this country (Damasio, 2010; Tamir & Mitchel, 2011). Presumably, we must be able to monitor and update our here-and-now on all these scales. However, spatial relations in small-scale scenarios might change more dynamically than in large-scale scenarios; for instance, we need to continuously update objects' positions when we move in a room (Avraamides & Carlson, 2003; de Vega & Rodrigo, 2001; Franklin & Tversky, 1990). Moreover, within small-scale scenarios, there are significant functional differences between the proximal or peripersonal space, which includes reachable objects, and the distal space including unreachable objects (Tamir & Mitchel, 2011; Costantini et al., 2010; Coello & Bonnotte, 2013). The embodied cognition approach has typically dealt with small-scale spatial information. For instance, action-related language, usually associated with motor processes, is framed in the peripersonal space where manipulation processes take place. By contrast, the objects at the spatial scale of geographical scenarios are typically non-manipulable and would not trigger any motor processes.

Furthermore, we only need to update our geographical here-and-now occasionally, when we travel long distances (e.g., to another country) or navigate from one town to another. These features of geographical scenarios open the possibility that understanding geographical space involves more abstract or disembodied representations than understanding small-scale layouts (Tamir & Mitchel, 2011). Despite that, this study found that some deictic verbs of motion could induce participants to activate their town and region of residence, providing evidence of self-centered geographical perspective, confirming and extending previous findings (de Vega et al., 2015).

Another important difference between the current study and other studies on language and spatial cognition is that the perspective effect occurs implicitly. In contrast, in most studies, the task demands explicit spatial representations. Thus, in those studies, participants are typically instructed to learn a layout (either from visual materials or from verbal descriptions) before performing a set of spatial judgments about the objects' spatial relations in the layout (e.g., Avraamides & Carlson, 2003; de Vega & Rodrigo, 2001; Franklin & Tversky, 1990). Conversely, the perspective effects reported here occurred spontaneously in the course of ordinary language comprehension, without any explicit perspective-taking instruction. In other words, the phenomena analyzed here are quite representative of online language comprehension, whereas the classical studies are more akin to learning processes of spatial information.

In a set of interesting studies, Fini et al., (2014, 2015) also tested implicit perspective-taking in extrapersonal space by a 3D virtual environment. They found that participants estimated that a target was closer when the reference was the virtual human agent rather than when it was a static object. Moreover, this effect was absent when the virtual agent was not free to move (Fini et al., 2015), suggesting that participants tend to spontaneously adopt another person's perspective when making judgments about their immediate environment. Notably, in another study, using tDCS, the authors identified that cathodal and anodal stimulation on the inferior frontal gyrus (a region associated with semantic categorization and interpersonal motor resonance mechanisms) modulated extrapersonal perspective-taking in front of agent / object stimuli (Fini et al., 2017). While stimulation results were ambiguous in agent perspective taking, it clearly impacted on object categorization (cathodal stimulation improved the distance recognition between objects, while anodal stimulation ameliorated it). Interestingly, anodal stimulation increased the capability of adopting an external agent perspective, especially in individuals with low scores on the perspective-taking scale of the IRI survey. In our study, we analyzed just how a paralinguistic cue could prompt geographical perspective taking. In the future, further experiments could be developed for analyzing brain mechanisms involved in processing this kind of cues and variables such as differences in perspective-taking scores.

The results of our experiment showed that participants listening to first-person deictic paragraphs rely on the regional accent as a cue to establish the deictic center. In previous studies with written narratives, the shift to other-person perspectives relied exclusively on linguistic features, such as the grammatical person or the amount of information provided about the protagonist and/or the narrative' deictic center. For instance, Brunyé et al. (2009) found that first-person short paragraphs induced self-perspective, whereas providing some information about the first-person narrator induced readers to shift to an external or other-person perspective. In the same way, García-Marco et al. (2016) provided readers with descriptions of fictional characters and their place of residence, and they found that readers were able to consistently take their perspective. However, this experiment shows for the first time that listeners can rely on a paralinguistic cue to shift from their own perspective to the other-person perspective. According to social psychologists, listeners can use regional accents to apply evaluative group stereotypes to the speakers (see Fuertes et al., 2012, for a meta-analysis). Here we demonstrated that, in the absence of any other relevant information, the regional accent also induces spatial perspective. Participants kept the default self-perspective when the speaker's accent was local, but they shifted to the other-person perspective when the speaker's accent was from another geographical region, demonstrating that a paralinguistic feature of speech (the regional accent) plays a role in the listener's perspective-taking.

Based on the interactive effects of regional accent and geographical place on coherence judgments, we concluded that listeners use “self-perspective” or shift to “another-person perspective”. Yet, an alternative interpretation is that participants’ coherence judgments rely on the probability or plausibility of the statements rather than on perspective-taking. In other words, participants would judge “come to Tenerife” spoken with local accent, or "come to Madrid" spoken with Castilian accent to be coherent, just because they are more probable or plausible. Undoubtedly, coherence judgments are technically probabilistic, since not all participants respond equally. As Fig. 1 shows, the ‘incoherent’ versions of the task were still considered ‘coherent’ by some participants. For instance, it makes sense, for a person living in Tenerife, to judge coherent "come to Tenerife" pronounced with Castilian accent, because he/she has come across tourists saying such utterance. What is clear is that to produce coherence judgments, the participants must be able to combine and assess two diverse sources of spatial information: one purely linguistic (the deictic verb and the geographical place in the sentence) and the other paralinguistic (the regional accent). As a general rule, statements in which the speaker’s regional accent matches the protagonist’s geographical location, tend to be more acceptable than those in which the accent and location do not match.

In future studies, it would be interesting to complement these results with finer-grained cognitive and neural measures, to reveal the mechanisms which combine deictic and accent information to compute geographical perspective. Meanwhile, it is important to mention that the geographical perspective hypothesis was also supported by prior EEG experiments, which identified the N400 component associated with congruence, induced by the deictic verb and the geographical place (de Vega et al., 2015; García-Marco et al., 2016). In those studies, participants read motion sentences similar to those used here either without an explicit deictic reference (de Vega et al., 2015) or with a character's brief narrative context (García-Marco et al., 2016). Interestingly, the results of both studies showed a geographical perspective effect: "coming to distant places" elicited larger N400-like component compared to “coming to close places” or “going to distant places”. In those experiments with written materials it could be argued that the geographical perspective effects were just "statistical", derived from the fact that some verb-place combinations may occur more frequently than others in the participants' environment. For instance, the participants could have been more often exposed to "I come to Tenerife" than to "I come to Madrid" (see de Vega et al., 2015, for a discussion of this issue). However, in the current experiment the perspective effects cannot be attributed to word co-occurrence or any other statistical feature of words. The experiment showed that less frequent utterances like "come to Madrid" or "bring to Tenerife" were accepted as coherent when pronounced with an appropriate regional accent.

## Conclusions

In sum, this study supports the idea that short narratives that include deictic markers such as first pronouns and proximal motion verbs prompt listeners to establish self-perspective in a similar way as face-to-face conversations. Coherence effects demonstrated the self-perspective: sentences describing proximal motions toward the listener's geographical place were better understood when the regional accent was aligned with the geographical place. This shifting to the other-person perspective can be induced by a regional accent, demonstrating its role as a powerful marker of the geographical deictic center. Further research will be needed to understand better how geographical self-perspective is implemented in the brain. For instance, neuroimaging experiments could explore the extent to which geographical self-perspective recruits the self-relevance neural network. Another possible research line could be to analyze how perspective induced by pronouns and deictic verbs differs between small-scale layouts and geographical scenarios. In any case, the experiments reported here provide a new approach to the functional interface between language and spatial cognition. Unlike classical paradigms that require learning complex spatial environments, we have shown how online understanding of minimal paragraphs with deictic markers induces powerful self-perspective anchored in the current geographical place.

## Data Availability

All the data can be consulted on the following link: https://osf.io/z5wdh/?view_only=f9696fc7bcd64db18cb5a4094b34268d.
